# Protocol of Co-Culture of Human Osteoblasts and Osteoclasts to Test Biomaterials for Bone Tissue Engineering

**DOI:** 10.3390/mps5010008

**Published:** 2022-01-14

**Authors:** Giorgia Borciani, Giorgia Montalbano, Nicola Baldini, Chiara Vitale-Brovarone, Gabriela Ciapetti

**Affiliations:** 1 Biomedical Science and Technologies Laboratory, IRCCS Istituto Ortopedico Rizzoli, 40136 Bologna, Italy; giorgia.borciani@ior.it (G.B.); nicola.baldini@ior.it (N.B.); gabriela.ciapetti@ior.it (G.C.); 2 Department of Applied Science and Technology, Politecnico di Torino, Corso Duca degli Abruzzi 24, 10129 Torino, Italy; giorgia.montalbano@polito.it; 3 Department of Biomedical and Neuromotor Sciences, University of Bologna, 40136 Bologna, Italy; 4 Laboratory of Nanobiotechnology, IRCCS Istituto Ortopedico Rizzoli, 40136 Bologna, Italy

**Keywords:** co-culture, osteoblast, osteoclast, human peripheral blood mononuclear cells, bone remodeling, crosstalk, biomaterial, scaffold, biocompatibility, bone tissue engineering

## Abstract

New biomaterials and scaffolds for bone tissue engineering (BTE) applications require to be tested in a bone microenvironment reliable model. On this assumption, the in vitro laboratory protocols with bone cells represent worthy experimental systems improving our knowledge about bone homeostasis, reducing the costs of experimentation. To this day, several models of the bone microenvironment are reported in the literature, but few delineate a protocol for testing new biomaterials using bone cells. Herein we propose a clear protocol to set up an indirect co-culture system of human-derived osteoblasts and osteoclast precursors, providing well-defined criteria such as the cell seeding density, cell:cell ratio, the culture medium, and the proofs of differentiation. The material to be tested may be easily introduced in the system and the cell response analyzed. The physical separation of osteoblasts and osteoclasts allows distinguishing the effects of the material onto the two cell types and to evaluate the correlation between material and cell behavior, cell morphology, and adhesion. The whole protocol requires about 4 to 6 weeks with an intermediate level of expertise. The system is an in vitro model of the bone remodeling system useful in testing innovative materials for bone regeneration, and potentially exploitable in different application fields. The use of human primary cells represents a close replica of the bone cell cooperation in vivo and may be employed as a feasible system to test materials and scaffolds for bone substitution and regeneration.

## 1. Introduction

The coupling of bone formation by osteoblasts (OBs) and bone resorption by osteoclasts (OCs) is at the base of the bone remodeling process and plays a crucial role in the maintenance of bone volume and skeletal homeostasis, with the crosstalk between OBs and OCs tightly regulated by systemic factors and molecules locally secreted [1]. Osteoprogenitors, osteocytes and bone lining cells are the other cells involved in the finely orchestrated process of bone tissue homeostasis, but OBs and OCs are considered the key players in the bone remodeling process and their coordinated activity has always been addressed in the frame of bone biology studies [2]. Alongside the study of pathophysiological mechanisms of the bone tissue, the OB/OC co-culture systems have been developed to test potential innovative materials for bone tissue engineering (BTE) approaches [3,4,5]. Indeed, many aspects can be investigated using the OB/OC co-culture system, since this model allows to recreate in vitro a bone microenvironment that more closely mimics the natural complex interactions between bone cells [6].

In this context, the use of human primary cells is strongly recommended for a reliable bone replicate in vitro, even if the poor availability of donor tissue, the phenotypic heterogeneity caused by donor variability, and the limited supply undermine their large application in the research field [7]. On the contrary, cell lines allow for large-scale expansion and reproducibility, but they represent a less reliable model. As remarked by Wilkesman et al., the standardization of experiments is facilitated by the use of cell lines instead of primary cells, since cell lines are easily expanded, with reproducible results and a more feasible comparison among different studies [8]. Even if cell lines show a higher replication rate, they share only partially the phenotype and properties of the primary cells [9,10]. Moreover, as underlined by Abdallah et al., “access to a standardized source of mature human OCs from peripheral blood mononuclear cells (PBMCs) is needed to analyze their roles in bone regeneration and repair” [11].

Therefore, the set-up of a well-designed and reliable human-derived OB/OC co-culture model is a milestone for further studies on innovative materials for BTE, for screening drug treatments, and for deciphering the basic mechanisms of bone metabolic diseases such as osteoporosis [12,13,14,15].

To date, a wide array of co-culture systems has been proposed and explored for different purposes: several authors reported examples of OB/OC co-culture to obtain mature and fully functional cells, by employing human bone primary cells [16], rodent or mammalian cell lines [17,18] or combinations of different cell types [19,20]. However, a widely accepted model of the in vitro reproduction of bone microenvironment is still lacking and a simple in vitro co-culture model to obtain mature OBs and OCs from human precursors has not yet been reported.

The complexity derived from the presence of more than one cell type, with different aspects and key design benchmarks to be considered within the single system, may represent a discouraging issue for researchers. Indeed, beyond the type of cells, several parameters need to be assessed and optimized, including the source of primary cells, the spatial and temporal seeding parameters, the ratio between the different cell types, the composition of the culture medium with or without supplements, and, not least, the static or dynamic system of culture [6].

In the present work, we developed a co-culture of human osteoblasts (OBs) derived from trabecular bone and human osteoclast precursors (PBMCs) isolated from the buffy coat, to set up a human bone cells system able to recreate in vitro a bone microenvironment. We describe the following steps with the aim to standardize the OB-PBMC system: (i) isolation and expansion of human trabecular bone-derived osteoblasts; (ii) isolation of monocytes, as osteoclast precursors, from a human donor-derived blood sample (buffy coat); (iii) co-culture of osteoblasts and osteoclast precursors in an indirect culture system, i.e., allowing their interaction through paracrine communication; (iv) testing new materials for BTE by using the present OB-PBMC co-culture system.

The model proposed attempts to reproduce in vitro the physiological system in which OBs and OCs exchange reciprocal signals, mutually influencing their differentiation. Even though a 2D in vitro experimental set-up cannot entirely mimic the in vivo condition, it may represent a first fundamental step for further studies.

Our indirect co-culture system can be thus applied during the screening of new potential bone substitute materials, where the tissue response and the potential interactions between material and native bone cells need to be specifically investigated. In order to disclose the cell response to the foreign surface, the biomaterial/scaffold under assay can be alternately placed in direct contact with OBs or PBMCs, having the other cell type in indirect contact, that is cell-cell communication by paracrine signaling mechanism. For instance, OBs can be seeded on the scaffold to evaluate the direct effect of material components on OBs, whereas PBMCs are maintained in the co-culture, thus allowing physiological paracrine signal exchange. Likewise, PBMCs can be seeded onto the material and OBs co-cultured, to estimate the direct effects onto PBMCs. In the two alternatives, the presence of both OBs and PBMCs is needed to assure the cross-talk between OBs and OCs, with the advantage of studying both types of cells in an “all in one system”. Thanks to the physical separation of OBs and PBMCs by means of a transwell device, the effects of materials on the morphology, behavior, and maturation of cells can be assessed for each cell type.

We applied our cell co-culture system to test a new smart material, which is a type I collagen-based hydrogel containing strontium-enriched mesoporous bioactive glass particles and rod-like hydroxyapatite nanoparticles.

Moreover, the developed model can be exploited in other research fields, such as for the in-depth analysis of signaling pathways between bone cells, for studies on the pathophysiological mechanisms of the bone tissue, and for screening drug therapies for bone-related diseases. For instance, in our model, human OBs obtained from patients suffering from age-related bone diseases could be used to evaluate the cellular changes induced by the disease, as well as to observe the potential effects of the smart material on the cell behavior.

Basically, the system herein proposed aims to provide guidelines for the preliminary evaluation of new materials for BTE approaches, with the awareness that further improvements may be achieved.

## 2. Experimental Design

**Figure 1:**
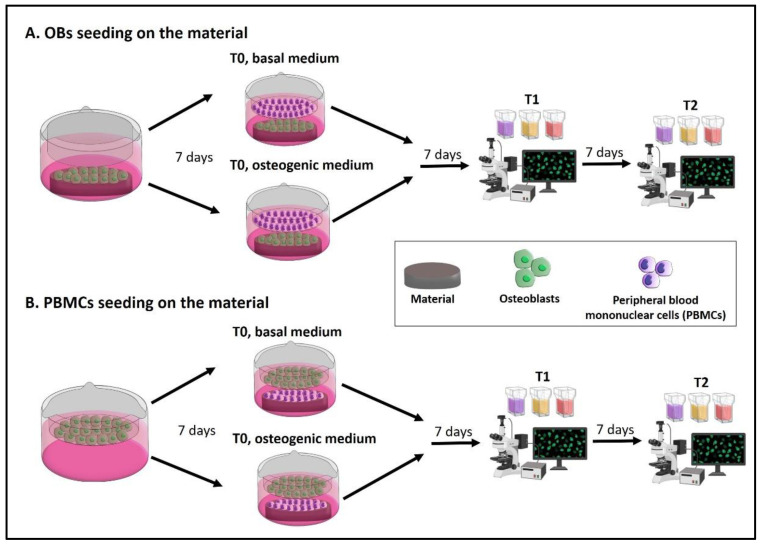
Experimental design of the protocol. Two types of indirect co-cultures can be set up, by choosing what cell type may be seeded on the material and which one may be co-cultured. (**A**) OBs on the material: OBs are seeded on the material and left 7 days until cell confluence. Then, PBMCs are seeded into the transwell and the indirect co-culture starts. (**B**) PBMCs on the material: OBs are seeded into the transwell and left 7 days until cell confluence. Then, PBMCs are seeded on the material and the indirect co-culture starts. After 7 days (T1) and 14 days (T2) the following assays are performed: Alizarin Red S, von Kossa and alkaline phosphatase (ALP) staining on OBs, while tartrate-resistant acid phosphatase (TRAP), Hoechst and phalloidin-tetramethylrhodamine B isothiocyanate (TRITC) or phalloidin-fluorescein isothiocyanate (FITC) staining are used on PBMCs.

The timeline and the main steps of the experimental plan are reported in Figure 1.

At first, isolate OBs from trabecular bone sample and expand, and isolate PBMCs from buffy coat. Then, two different options can be performed to test a new material with this protocol:

**A.**  The seeding of OBs on the material with PBMCs in the indirect co-culture. Seed OBs on the material and wait 7 days until cell confluence, then seed PBMCs into the transwell to begin the indirect co-culture (T0). The indirect co-culture is maintained in osteogenic medium or basal medium for 14 days (T2), with the intermediate step of 7 days (T1) to check cell behavior.
**B.**  The seeding of PBMCs on the material with OBs in the indirect co-culture. Seed OBs into the transwell and wait 7 days until cell confluence, then seed PBMCs on the material to begin the indirect co-culture (T0). The indirect co-culture is maintained in osteogenic medium or basal medium for 14 days (T2), with the intermediate step of 7 days (T1) to check cell behavior.

### 2.1. Materials: Reagents and Reagents Set Up (for Composition of Solutions, See Section 5) (All the Solutions, Instruments and Tissue Culture Plastics Must Be Sterile)

- Wash buffer: 1× phosphate buffered saline (PBS).
- Fixative solution n.1.
- Fixative solution n.2.
- Fixative solution n.3.
- Fixative solution n.4.
- Ficoll-Paque PLUS.
- Ca^+2^-free complete culture medium for OBs.
- Complete culture medium for OBs.
- Complete culture medium for PBMCs.
- Cell freezing medium.
- Basal medium.
- Osteogenic medium.
- Differentiation medium for PBMCs.
- Trypsin-EDTA solution.
- Alizarin Red S (ARS) staining solution.
- Von Kossa staining solution.
- Fluorescein isothiocyanate (FITC)-phalloidin (Sigma-Aldrich, P-5282, Milan, Italy) staining solution.
- Leukocyte Alkaline phosphatase assay (ALP staining) (Sigma-Aldrich, 86R, Milan, Italy).
- Acid phosphatase leukocyte assay (Tartrate-resistant acid phosphatase—TRAP staining) (Sigma-Aldrich, 387A, Milan, Italy).
- Nuclear staining.
- Erythrosin B staining solution 1×. Instead of Erythrosin B, Trypan blue dye may be applied. Please note that the operating principle is the same: both dyes stain dead cells only, since the dyes cannot penetrate the cell membrane of live cells to enter the cytoplasm. Under light microscopy, count only non-stained cells (viable).
- EDTA-based buffer for the lysis of red blood cells (RBCs).
- Tris buffer + 0.1% Triton X-100 pH 7.5.
- P-nitrophenyl phosphate liquid substrate system (PNP) (Sigma-Aldrich, N7653, Milan, Italy).
- 10% cetylpyridinium solution.
- 0.1 M sodium cacodylate buffer (Thermo Fisher Scientific, 15463149, Milan, Italy).
- 1% osmium tetroxide solution.
- Live dead assay (LIVE/DEAD™ Cell Imaging Kit (488/570), Invitrogen, Thermo Fisher Scientific, Milan, Italy).

### 2.2. Equipment

- Tissue culture plastic 25 cm^2^ and 75 cm^2^ flask for isolation and expansion of OBs, (75 cm^2^ Euroclone, ET7076, Milan, Italy; 25 cm^2^ Corning, Life Sciences, Falcon, 353108, Turin, Italy).
- 8-well glass chamber-slides for PBMCs and OBs single-culture (SARSTEDT, 94.6170.802, Nümbrecht, Germany), 0.8 cm^2^ growth area.
- Tissue culture plastics 24-well plate for the co-culture (Falcon, 353047, Turin, Italy).
- Transwell device: insert for 24-well tissue culture plate, with polyethylene terephthalate (PET) membrane and polystyrene (PS) as frame material, transparent, sterile, non-pyrogenic/endotoxin-free, non-cytotoxic, with a flat base, 0.4 µm pore size, 0.3 cm^2^ growth area, 0.1–0.4 mL working volume (SARSTEDT, 83.3932.041, Nümbrecht, Germany). Please note that is important that the transwell fit into the well of the 24-well tissue culture plates and the plate can be closed.
- Tissue culture (TC) coverslip for 24-well tissue culture plates (13 mm diameter): polyethylene terephthalate, glycol-modified (PET-G), transparent, sterile, non-pyrogenic/endotoxin-free, non-cytotoxic (SARSTEDT, 83.1840.002, Nümbrecht, Germany).
- Horizontal laminar flow station (Heraguard, Thermo Fisher Scientific, Milan, Italy).
- CO_2_ incubator.
- 50-mL Falcon tubes.
- Pipettes.
- p200 and p1000 pipet tips.
- Centrifuge.
- Fluorescent optical microscope (Nikon Eclipse E800) equipped with NIS-Element image software BR4.00.00 (Nikon), NIS-Element image software Advanced Research (Version 5.30) (Nikon, Amsterdam, Netherlands).
- Inverted phase contrast microscope.
- Camera (microscope-connected camera).
- Image analysis software.
- Hemocytometer.
- Spectrophotometer for microplates (Infinite F200 PRO, TECAN, Mannedorf, Switzerland).

## 3. Procedure

### 3.1. Osteoblasts Isolation Procedure

Isolate osteoblasts (OBs) from trabecular bone samples of healthy patients undergoing surgery for fracture repair or other orthopedic procedures, following patients’ informed consent approved by the Institutional Ethical Committee.

Caution: bone sample collection should always comply with national and institutional laws and regulations. Informed consent must be obtained from patients.

Once removed the soft tissue with a scalpel, proceed to isolate OBs by single-cell sprouting from bone fragments:

(a) Wash bone fragments in PBS for 5 min twice and place them in 25 cm^2^ culture flask with at least 5 up to 10 mL of Ca^+2^-free complete culture medium for OBs (to inhibit fibroblast growth). Culture OBs at 37 °C, 5% CO_2,_ and 95% humidity with Ca^+2^-free complete culture medium changes every 3 days.
 
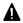
**CRITICAL STEP**: use a minimum volume of medium in order to maintain the bone fragments covered by medium but adherent to the flask bottom.
(b) At confluence (usually after 2–3 weeks), wash OBs with PBS, detach them from the flask by trypsin-EDTA digestion, and subculture by reseeding in two new 75 cm^2^ flasks. At confluence OBs may be detached and used (passage 1).
(c) Count OBs: stain 20 μL of cell suspension with 20 μL of erythrosine B solution, mix gently, and collect 10 μL to be counted in hemocytometer under the inverted microscope.

Please note that OBs in excess may be stored in liquid nitrogen and used after thawing for further experiments:

I. To store OBs in liquid nitrogen, centrifuge the residual OBs at 1050 rpm (RCF 132) for 30 min (Centrifuge Eppendorf 5810). Resuspend the pellet in 1 mL of cell freezing medium and transfer the cell suspension into a cryogenic vial. Use 1 mL of cell freezing solution for each 25 cm^2^ or 75 cm^2^ flask.
II. Keep the cryogenic vial at −80 °C for 3 days.
III. Store the cryogenic vial in liquid nitrogen.

### 3.2. Peripheral Blood Mononuclear Cells Isolation Procedure

Isolate peripheral blood mononuclear cells (PBMCs) from buffy coats of healthy donors following the Ficoll-Paque density-gradient method.

Caution: blood sample collection should always comply with national and institutional laws and regulations. Informed consent must be obtained from patients.

**Figure 2:**
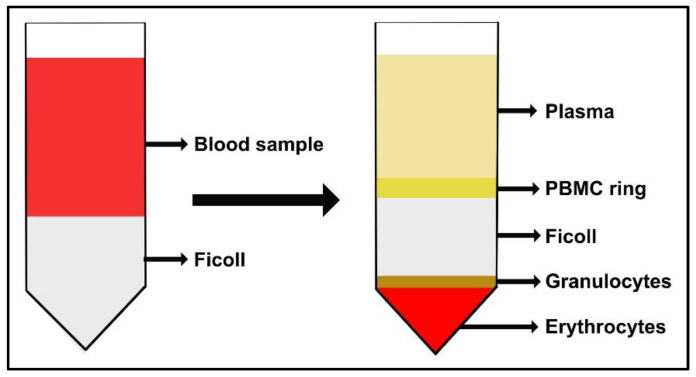
Schematic representation of the buffy coat sample stratified on Ficoll-Paque PLUS. After subsequent density gradient centrifugations, the PBMC ring layer is collected.

(a) Dilute the buffy coat sample with an equal amount of 1× PBS (1:1 final dilution).
(b) Pour 3 mL of Ficoll-Paque PLUS at the bottom of 15 mL tubes. Subsequently gently add 4 mL of diluted blood overlaying the Ficoll layer, being careful not to break the gradient layer. When working with 50 mL tubes, the Ficoll:blood ratio is usually 15:20 mL; these volumes may be increased, but the efficiency of PBMC isolation can be negatively affected.
(c) Gradient centrifugation: centrifuge the tubes at room temperature (RT) and 2000 rpm (RCF 479) for 30 min with the breaks off.
 
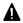
**CRITICAL STEP** turning off the break enables the PBMCs to stay onto the gradient layer without being forced into the erythrocytes layer, while keeping the break on during the deceleration could disrupt the density gradient since a rapid and turbulent vortex is generated in the middle of the tube. This vortex picks up the thin layer of cells and redistributes them in the supernatant.
(d) At the end of the first gradient centrifugation, PBMCs form a “ring” between Ficoll and plasma, as illustrated in Figure 2. Harvest each layer of PBMCs and transfer it into a new tube. Then, add 15 mL (or 50 mL) of PBS.
 
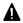
**CRITICAL STEP** harvest the PBMCs ring with a 1000 µL pipette tip.
(e) Centrifuge the tubes at RT and 1600 rpm (RCF 306) for 20 min keeping the breaks off.
(f) Harvest each PBMC-pellet and transfer to new tubes. Then, add 15 mL (or 50 mL) of PBS.
 
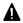
**CRITICAL STEP** harvest the PBMCs ring with a 1000 µL pipette tip.
(g) Centrifuge the tubes at RT and 1600 rpm (RCF 306) for 10 min keeping off the breaks.
(h) Harvest each pellet and transfer into a new tube. Then, add 15 mL (or 50 mL) of PBS.
 
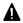
**CRITICAL STEP** harvest the PBMCs ring with a 1000 µL pipette tip.
(i) Centrifuge the tubes at RT and 1400 rpm (RCF 234) for 10 min keeping the breaks off.
(j) Remove the supernatant and resuspend the pellet in DMEM-HG (about 30 mL).
(k) Count PBMCs: dilute 50 μL of cell suspension in 450 μL of PBS, then mix gently, stain with 500 μL of 1× erythrosin B staining solution and mix gently again. Count the viable cells (viable cells are not pink-stained) in 10 μL of cell suspension in a hemocytometer under the inverted microscope.

Please note that red blood cells (RBCs) may be present in the cell suspension depending on the quality of PBMC isolation. If the number of RBCs makes it difficult to count PBMCs, RBCs may be lysed using an EDTA-based buffer.

### 3.3. Indirect OBs/PBMCs Co-Culture and Single-Culture Controls

**Figure 3:**
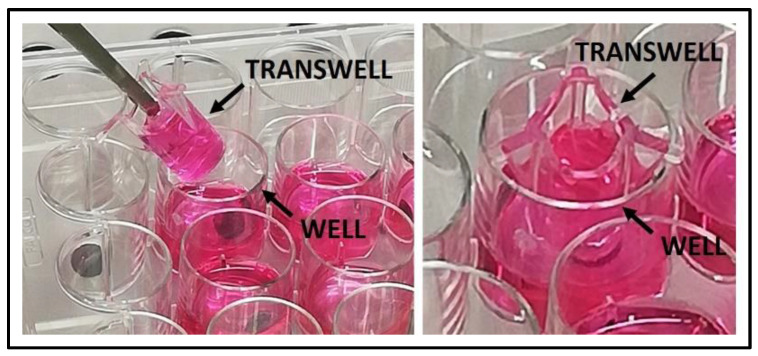
The set-up of the indirect co-culture with well and transwell device. The transwell has to fit perfectly into the well allowing the plate lid to close. Tweezers are used to remove the transwell from the well.

The assembly of the co-culture system is reported in Figure 3.

(a) Seed 5 × 10^3^ OBs in complete DMEM-LG into transwells seven days before the start of the co-culture, to achieve a confluent monolayer at day 7. Change the medium twice a week.
(b) Equip the bottom of the multiwell plate with a proper-sized TC coverslip previously pre-wetted with complete DMEM-HG for 2 h at least.
(c) Seed 6 × 10^6^/well PBMCs in complete DMEM-HG freshly isolated from buffy coat samples in the proper-sized TC coverslip at the bottom of the 24-multiwell plate, starting the co-culture (time 0).
(d) Incubate at 37 °C, 5% CO_2_ and 95% humidity to allow cell settling; the following day remove the old medium and refresh with the basal medium or osteogenic medium, with medium change twice a week.
(e) Maintain the indirect co-culture for 7 and 14 days in basal medium or osteogenic medium.

Please note that the indirect co-colture is maintained in culture medium without exogenous osteoclastogenic inducers in order to prompt the differentiation of PBMCs towards OCs only by means of the paracrine signals released by OBs. However, also some material leachable could affect osteoclast differentiation.

At the same time, to set up the cell culture controls, prepare OBs single-culture and PBMCs single-culture as differentiation controls, using 8-well glass chamber-slides, as follows.

For OBs single-culture:

(a) Seed 1.5 × 10^4^/well OBs in complete DMEM-LG in 8 well chamber-slides.
(b) Incubate at 37 °C to allow cell settling; the following day remove the old medium and refresh with the basal medium or osteogenic medium, with medium change twice a week.
(c) Maintain OBs in culture in basal and osteogenic medium, to be tested at 7 and 14 days.

For PBMCs single-culture:

(a) Seed 2.4 × 10^6^/well PBMCs in complete DMEM-HG in 8 well chamber-slides.
(b) Incubate overnight at 37 °C, 5% CO_2_ and 95% humidity to allow cell settling; the following day, remove the non-adherent PBMCs by changing the old medium with the differentiation medium for PBMCs. Refresh the medium twice a week.
(c) Maintain PBMCs culture in differentiation medium to be tested at 7 and 14 days..

### 3.4. Cell Biochemistry—Cytochemical Staining Protocols

At each endpoint, that is 7 (T_1_) and 14 days (T_2_):

(a) Remove the medium and separate the transwell (with OBs seeded) and the TC coverslip (with PBMCs seeded) of the same well and rinse the cell layers with PBS.
(b) For each cytochemical staining, fix the cell layer with the appropriate fixative solution as follows:
  I. For Alizarin Red S staining use 3.7% paraformaldehyde solution in PBS 1× for 20 min at RT.
  II. For von Kossa staining use ice-cooled methanol kept at −20 °C for 5 min.
  III. For Alkaline phosphatase (ALP) staining use fixative solution n.3 for 30 min at RT.
  IV. For tartrate-resistant acid phosphatase (TRAP) staining use fixative solution n.2 for 20 min.
  V. For phalloidin-tetramethylrhodamine B isothiocyanate (TRITC) or phalloidin-fluorescein isothiocyanate (FITC) use fixative solution n.1 for 5 min.
(c) Wash the cell layer with PBS 1× for 5–10 min for ARS, von Kossa, TRAP, and phalloidin stainings or distilled water for 45 min for ALP staining.
(d) Stain the cell layer.
  I. For Alizarin Red S staining: add Alizarin Red S staining solution for 1 h at RT.
  II. For Von Kossa staining: add 2% AgNO3 solution for 1 h at RT under light and wash twice with distilled water. Add 2.5% sodium thiosulfate solution for 5 min and wash twice with distilled water. Lastly, add 0.33% neutral red solution for 1 h. The different solutions must be added in the order reported.
  III. For Alkaline phosphatase (ALP) staining: add ALP stain solution for 15 min at RT avoiding light exposure.
   
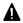
**CRITICAL STEP** Incubate the staining solution in dark conditions to minimize potential artifacts.
  IV. For tartrate-resistant acid phosphatase (TRAP) and Hoechst staining: add HEPES-Triton solution for 5 min to permeabilize cell membrane, wash with PBS; incubate cell monolayer with TRAP staining solution for 1 h at 37 °C in dark conditions.
   
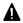
**CRITICAL STEP** Incubate the staining solution at 37 °C in dark conditions to minimize potential artifacts.
   After a fast rinse with distilled water, stain nuclei with Hoechst staining for 10 min in the dark.
   
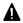
**CRITICAL STEP** Incubate the staining solution in dark conditions to minimize potential artifacts.
  V. For phalloidin-tetramethylrhodamine B isothiocyanate (TRITC) or phalloidin-fluorescein isothiocyanate (FITC): add TritonX 0.5% in PBS for 10 min to permeabilize cell membrane, incubate cell monolayer with phalloidin-FITC or phalloidin-TRITC staining solution in PBS for 45 min in the dark. After a fast rinse with PBS, stain nuclei with Hoechst staining for 10 min in the dark.
   
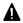
**CRITICAL STEP** Incubate the staining solution in dark conditions to minimize potential artifacts.
(e) After staining, wash cells for 5–10 min with PBS 1× in the case of ALP or with distilled water when using von Kossa, TRAP, phalloidin, and Alizarin Red S stainings; for Alizarin Red S staining rinse with distilled water until the washing solution becomes clear.
(f) Mount glass coverslip using 70% glycerol solution or specific aqueous mounting media.

**Figure 4:**
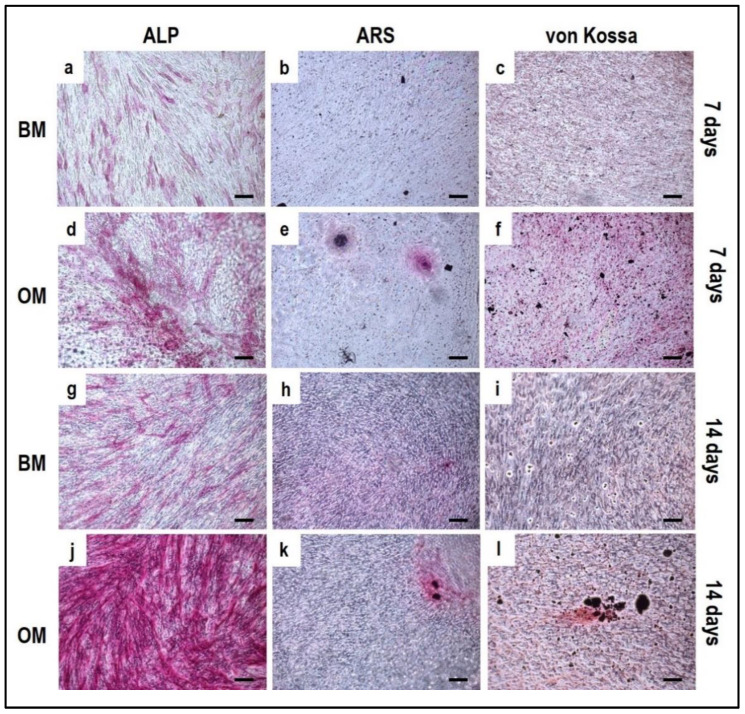
Microphotographs of alkaline phosphatase (ALP) (**a**,**d**,**g**,**j**), Alizarin Red S (ARS) (**b**,**e**,**h**,**k**) and von Kossa (**c**,**f**,**i**,**l**) stainings of OBs derived from the indirect co-culture in basal medium (BM) and osteogenic medium (OM) at 7 and 14 days. (**a**–**c**): basal medium at 7 days, (**d**–**f**): osteogenic medium at 7 days, (**g**–**i**): basal medium at 14 days, (**j**–**l**): osteogenic medium at 14 days. Scale bar: 100 µm.

**Figure 5:**
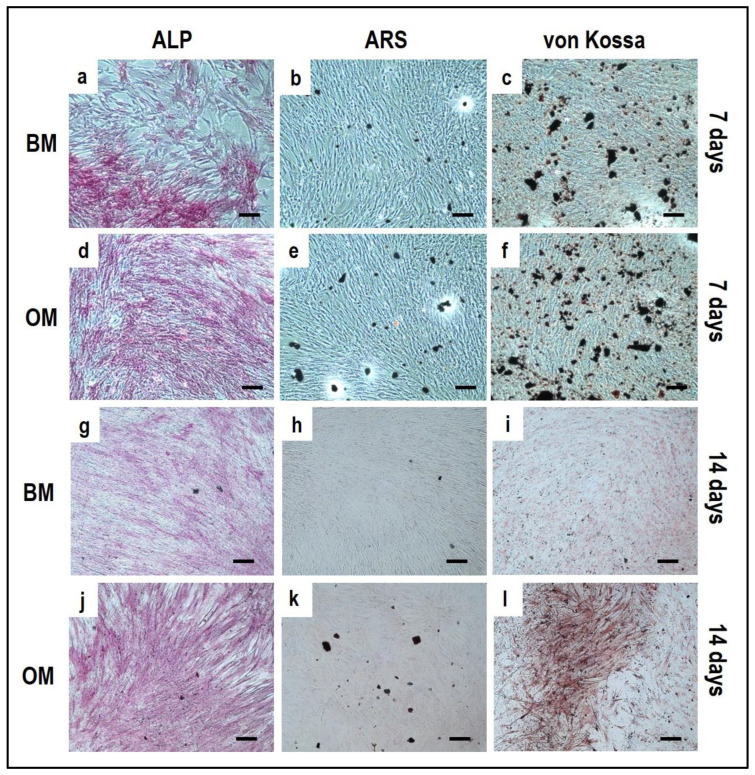
Microphotographs of alkaline phosphatase (ALP) (**a**,**d**,**g**,**j**), Alizarin Red S (ARS) (**b**,**e**,**h**,**k**) and von Kossa (**c**,**f**,**i**,**l**) stainings of OBs control culture at 7 and 14 days. (**a**–**c**): basal medium at 7 days, (**d**–**f**): osteogenic medium at 7 days, (**g**–**i**): basal medium at 14 days, (**j**–**l**): osteogenic medium at 14 days. Scale bar: 100 µm.

Alizarin Red S, von Kossa and alkaline phosphatase stainings applied to OBs from the indirect OBs/PBMCs co-culture (Figure 4) and from the control culture (Figure 5).

**Figure 6:**
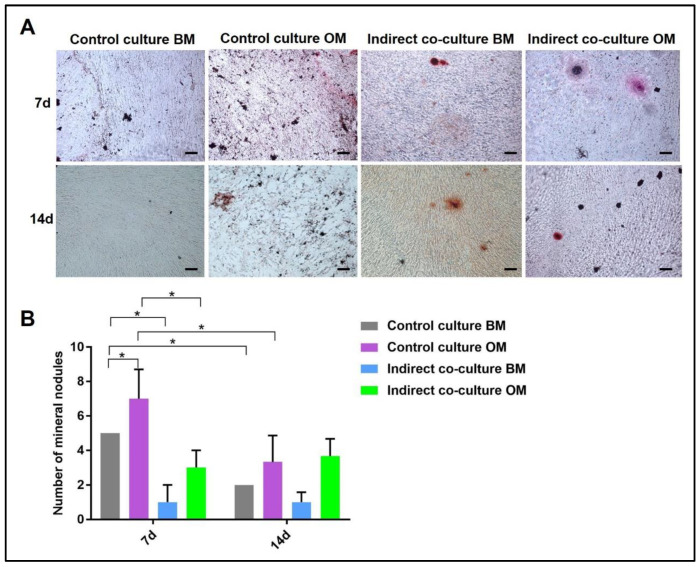
Quantification of mineral nodules deposited by OBs after Alizarin Red staining (ARS) in single-cultures (control culture) and indirect co-cultures exposed to basal medium (BM) or osteogenic medium (OM) for 7 and 14 days. In (**A**) representative fields of samples stained with ARS and in (**B**) the diagram of the data collected (* *p*-value ≤ 0.05). Scale bar: 100 µm.

**Figure 7:**
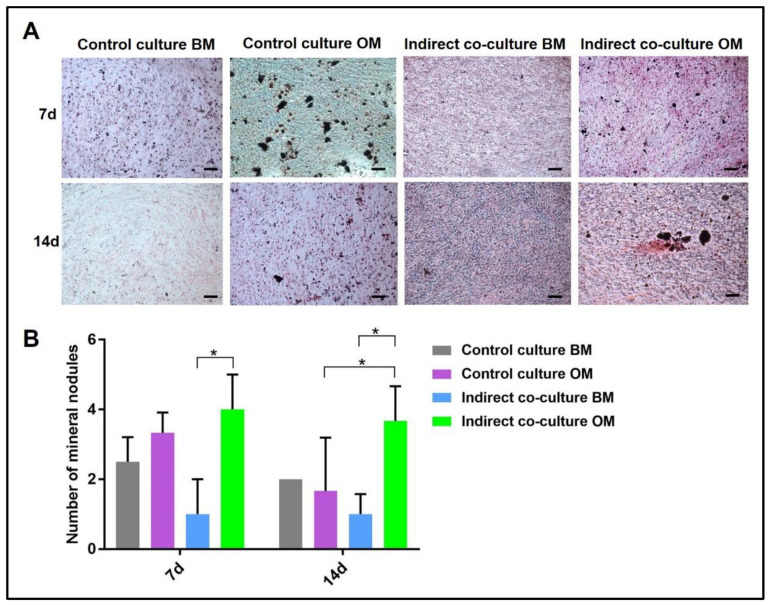
Quantification of mineral nodules deposited by OBs after von Kossa staining of single-cultures (control culture) and indirect co-cultures exposed to basal medium (BM) or osteogenic medium (OM) for 7 and 14 day. In (**A**) representative fields of samples stained with von Kossa and in (**B**) the diagram of the data collected (* *p*-value ≤ 0.05). Scale bar: 100 µm.

Concerning Alizarin Red and von Kossa stainings, the mineral nodules in osteogenic medium and basal medium were manually counted and compared to the controls, as reported in Figure 6 and Figure 7, respectively. The analysis was performed by light microscopy on three fields at 10× magnification.

**Figure 8:**
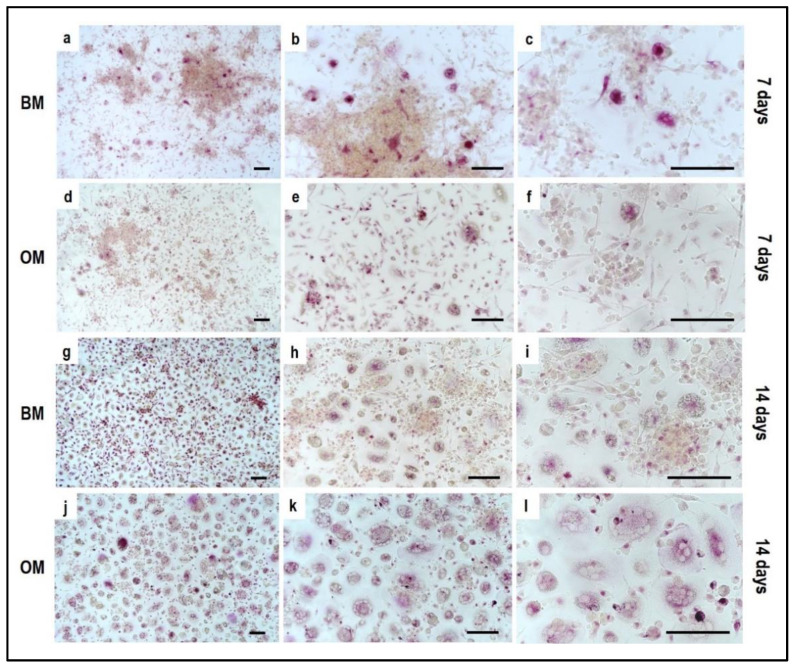
Microphotographs of Tartrate-Resistant Acid Phosphatase (TRAP) staining of PBMCs derived from the indirect co-culture in basal medium (BM) and osteogenic medium (OM) at 7 days and 14 days; (**a**–**c**): basal medium at 7 days, (**d**–**f**): osteogenic medium at 7 days, (**g**–**i**): basal medium at 14 days, (**j**–**l**): osteogenic medium at 14 days. Scale bar: 100 µm.

**Figure 9:**
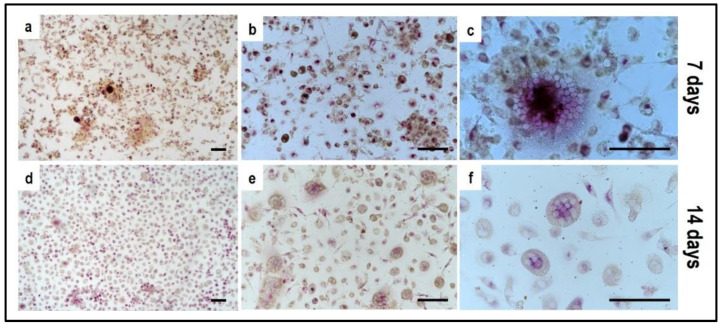
Microphotographs of PBMCs derived from the single-culture induced with M-CSF and RANKL, stained for Tartrate-Resistant Acid Phosphatase (TRAP) at 7 and 14 days. (**a**–**c**) control culture at 7 days, (**d**–**f**) control culture at 14 days. Scale bar: 100 µm.

**Figure 10:**
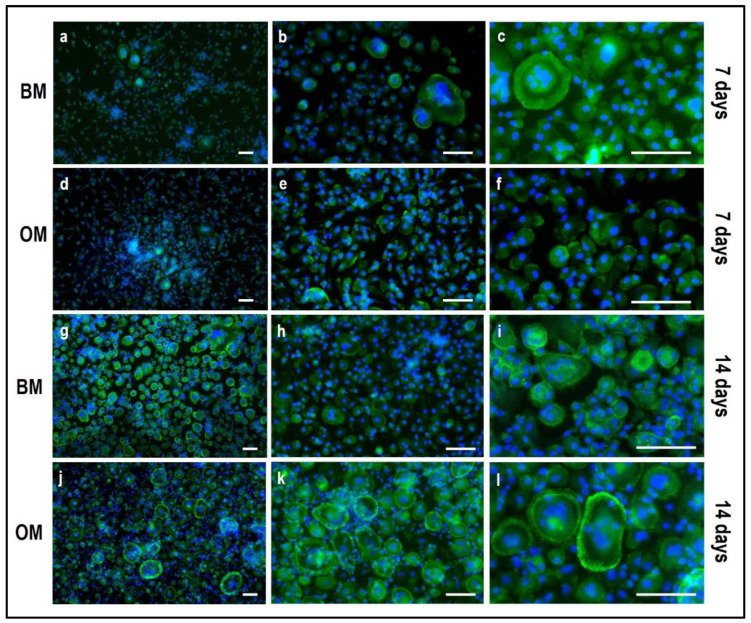
Microphotographs of PBMCs stained for multinuclearity (Hoechst dye, blue) and actin filaments/ring (Phalloidin-FITC, green) derived from the indirect co-culture in basal medium (BM) and osteogenic medium (OM) at 7 and 14 days. (**a**–**c**): basal medium at 7 days, (**d**–**f**): osteogenic medium at 7 days, (**g**–**i**): basal medium at 14 days, (**j**–**l**): osteogenic medium at 14 days. Scale bar: 100 µm.

**Figure 11:**
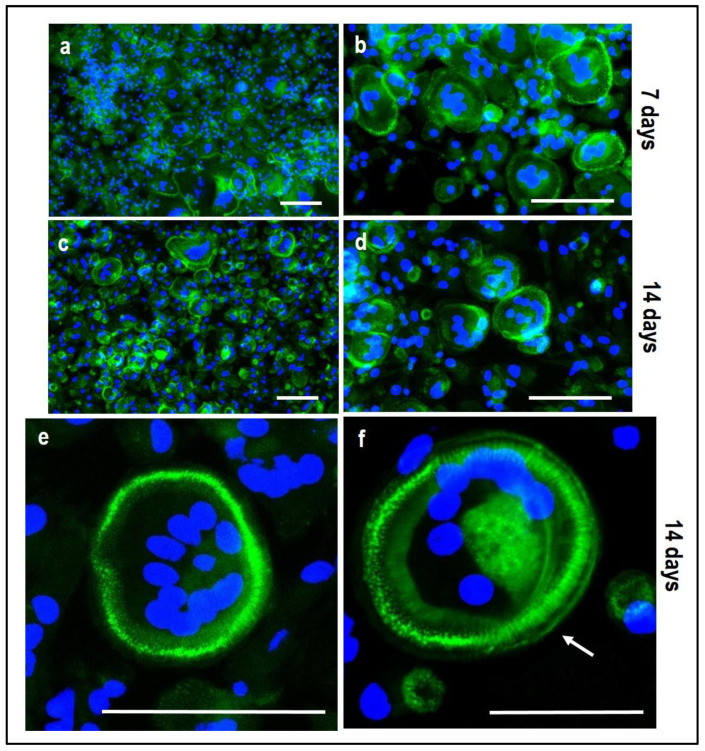
Microphotographs of PBMCs derived from the single-culture induced with M-CSF and RANKL, stained for multinuclearity (Hoechst dye, blue) and actin filaments/ring (Phalloidin-FITC, green) at 7 and 14 days. (**a**,**b**): single-culture (control culture) at 7 days, (**c**,**d**): single-culture at 14 days, (**e**,**f**): details of single-culture at 14 days. Scale bar: 100 µm.

Tartrate-resistant acid phosphatase (TRAP) and phalloidin-fluorescein isothiocyanate (FITC) stainings performed on PBMCs derived from the indirect OBs/PBMCs co-culture are shown in Figure 8 and Figure 9, while on PBMCs from the the control culture in Figure 10 and Figure 11, respectively.

**Figure 12:**
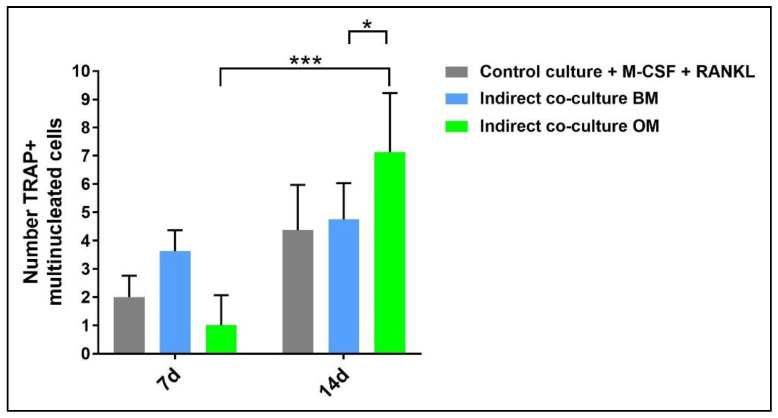
Diagram of the number of PBMCs positive for tartrate-resistant acid phosphatase derived from the indirect co-culture in basal medium (BM) or osteogenic medium (OM) at 7 days and 14 days compared to the control sample (CTRL + M-CSF + RANKL) (* *p*-value ≤ 0.05, *** *p* ≤ 0.001).

In addition, TRAP-positive multinucleated giant cells in the osteogenic medium and basal medium were manually counted and compared to the control (Figure 12). The analysis was performed by light microscopy on ten fields at 20× magnification.

**Figure 13:**
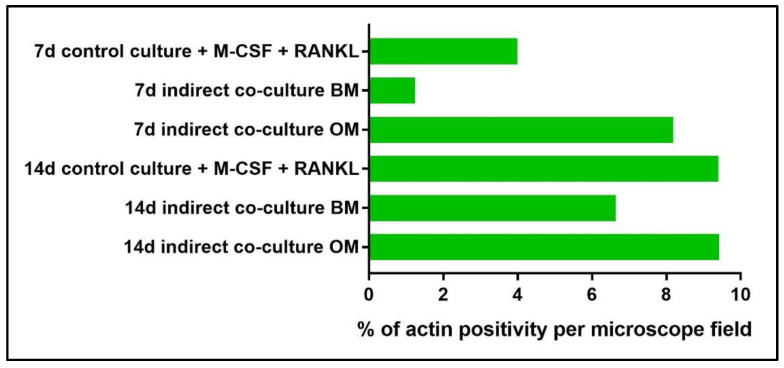
Diagram representing the percentage of area positively stained for actin filaments of PBMCs in both the single-cultures (control culture) and in indirect co-cultures exposed to basal medium (BM) or osteogenic medium (OM) for 7 and 14 days.

Moreover, actin filaments were quantified by image analysis using the NIS-Element imaging software Advanced Research (Figure 13). The threshold for the detection of the signal was set up at 38–255 with Clean 1× (Applications → Cell Counting). The analysis was performed by light microscopy on three fields at 10× magnification.

### 3.5. Material Testing with the Indirect Co-Culture

Before starting the indirect co-culture with the material, some considerations have to be done. In our case the material was prepared completely under sterile conditions. Otherwise, the material may be sterilized by immersion for at least 30 min up to 2 h in EtOH 70% or by UV-irradiation, depending on the composition of the material and the consequent reactivity to the sterilization method. Once performed this first step, material pre-wetting has to be accomplished as a pre-requisite for cell attachment:

(a) The material is dipped in complete culture medium for OBs or PBMCs: the presence of FBS in the medium, whatever it is, is needed for the adsorption of serum proteins to the material.
(b) Incubate at 37 °C overnight.
(c) Remove the complete culture medium.

The pre-wet material can be introduced in the indirect co-culture. As already described, two types of indirect co-cultures can be set up, by choosing what cell type may be seeded on the material and which one may be co-cultured.

Seeding OBs on the material and indirect co-culture with PBMCs:

(a) Prepare the OB suspension according to the material surface area and the seeding density/cm^2^ reported in Section 3.3—“a” (5 × 10^3^/0.3 cm^2^). To cover the entire surface with the cell suspension, resuspend the final number of OBs in a proper medium volume using the complete culture medium for OBs.
(b) Incubate the OB-laden material for 7 days to achieve a confluent monolayer, with medium change every 3 days.
(c) Seed PBMCs in the transwell with a cell seeding density as reported in Section 3.3—“c” (9 × 10^5^ PBMCs/transwell (3 × 10^6^/cm^2^)). Use complete culture medium for PBMCs.
(d) Incubate the PBMC-loaded transwell for 3 h at 37 °C.
(e) Insert the transwell in the well using sterile tweezers.
(f) Add basal medium or osteogenic medium both into the well and transwell.

Seeding PBMCs on material and indirect co-culture with OBs:

(a) Seed OBs in the transwell with a density as reported in Section 3.3—“a”, that is 5 × 10^3^ OBs/transwell (5 × 10^3^/0.3 cm^2^) using complete culture medium.
(b) Incubate OBs for 7 days to achieve a confluent monolayer, with medium change every 3 days.
(c) Prepare the PBMC suspension according to the material surface area and the seeding density/cm^2^ reported in Section 3.3—“c” (3 × 10^6^/cm^2^). To cover the entire surface with cell suspension resuspend the final number of PBMCs in a proper medium volume using the complete culture medium for PBMCs.
(d) Incubate the PBMCs-laden material for 3 h at 37 °C.
(e) Insert the OBs-loaded transwell into the well using sterile tweezers.
(f) Add basal medium or osteogenic medium to the well and transwell.

At 7 and 14 days proceed with Section 3.4—“Cell Biochemistry—Cytochemical staining protocols” to stain OBs and PBMCs.

If the material is translucent and bidimensional such as a hydrogel, an electrospun-layer, or a polymeric membrane, the cytochemical stains described in this protocol can be performed without additional precautions.

Otherwise, if the material is non-translucent, some morphological assays, including ALP or Alizarin Red staining for OBs and TRAP staining for OCs, have to be replaced by biochemical procedures giving quantitative results. Some of the assays used for the indirect co-culture with two collagen-based materials added with mesoporous bioactive glass particles (Coll_MBG) and nano-hydroxyapatite (Coll_HA) are reported underneath. These assays have been used for the 2D collagen-based hydrogels non-translucent due to the presence of genipin (blue-violet dye) as a crosslinker [21].

**Figure 14:**
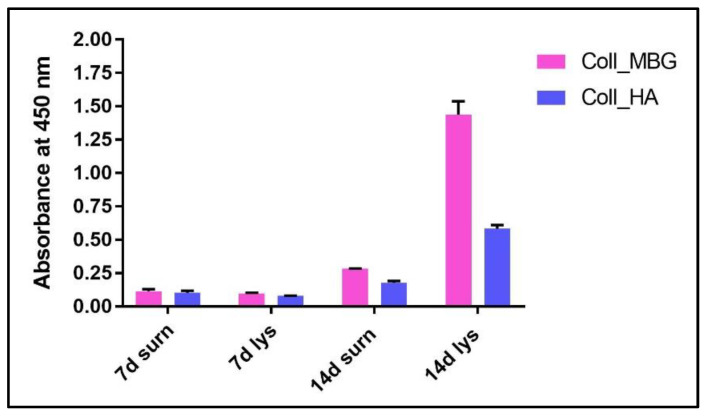
ALP quantification in supernatants and cell lysates of osteoblasts seeded on collagen-based scaffolds with nano-hydroxyapatite (Coll_HA) and collagen-baseds scaffold containing mesoporous bioactive glasses (Coll_MBG).

**Figure 15:**
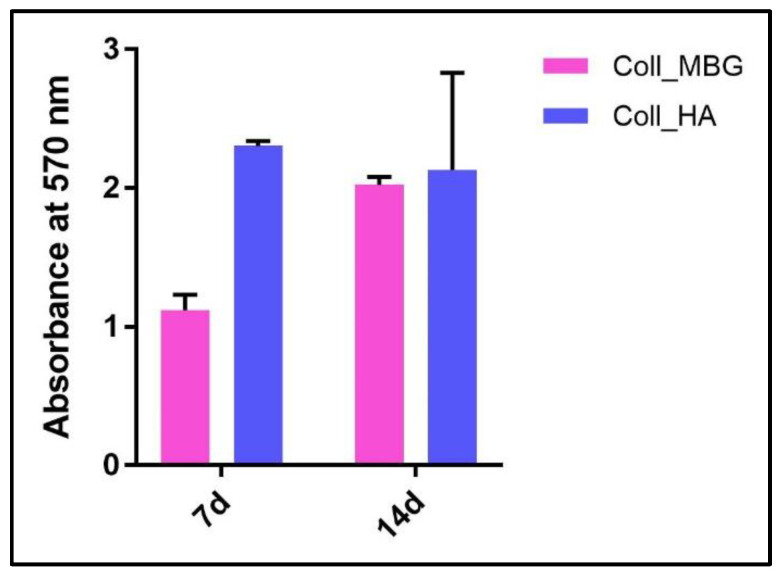
Alizarin Red eluate quantification after staining of osteoblasts seeded on the collagen-based scaffolds with nano-hydroxyapatite (Coll_HA) and collagen-based scaffolds containing mesoporous bioactive glasses (Coll_MBG).

**Figure 16:**
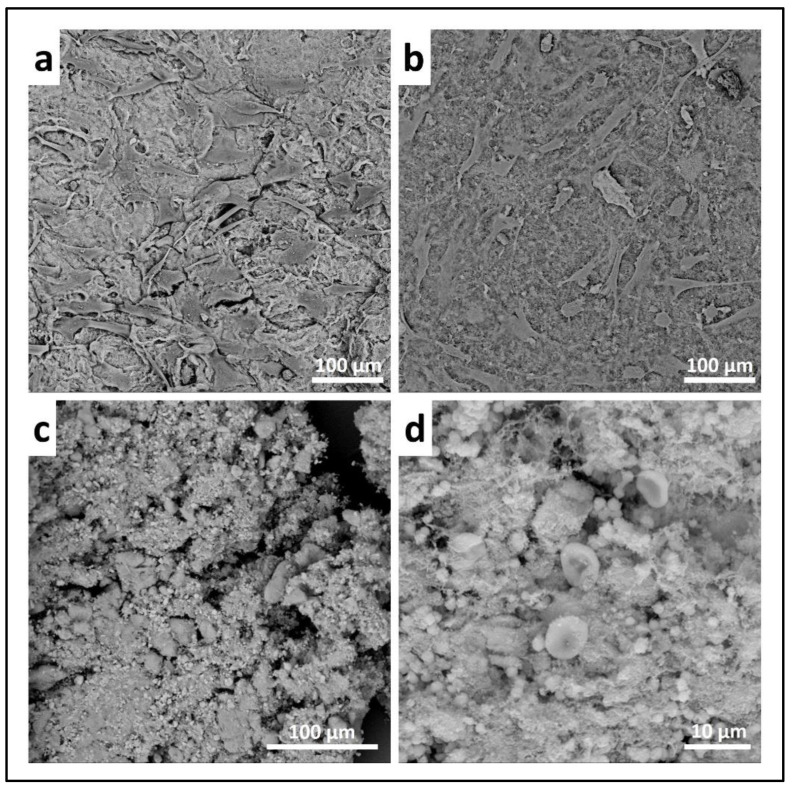
Scanning electron micrographs of human osteoblasts (**a**,**b**) and PBMCs (**c**,**d**) on collagen-based scaffolds containing mesoporous bioactive glasses (Coll_MBG) (**a**,**c**) and nano-hydroxyapatite (Coll_HA) (**b**,**d**) tested in co-culture, at 14 days.

**Figure 17:**
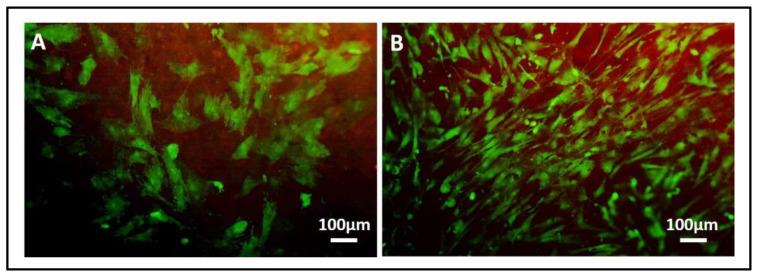
Representative images of osteoblasts seeded on collagen-based scaffolds containing mesoporous bioactive glasses (Coll_MBG) (**A**) and nano-hydroxyapatite (Coll_HA) (**B**) after live-dead staining at 7 days.

(a) ALP staining can be replaced by ALP quantification in the OB lysates and cell culture supernatant.
 To measure ALP in the cell lysate, after the removal of the cell culture medium, wash the sample twice with PBS and add 200 µL of Tris buffer + 0.1% Triton X-100 pH 7.5. Following incubation for 10 min at 37 °C, mix 100 µL of the cell lysate with 100 µL of PNP (final volume 200 µL). Transfer 100 µL of the mixture in a well of a 96 well-plate and read the absorbance at λ 405 nm with a microplate spectrophotometer.
 To measure ALP in the supernatant, harvest 100 µL of 3 day-aged cell culture medium for each sample and add 100 µL of PNP (final volume 200 µL). After transferring 100 µL of the mixture in a 96 well-plate, the absorbance is read at λ 405 nm with a microplate spectrophotometer.
 An example of ALP content in the OB lysate and supernatant derived from the indirect co-culture within Coll_MBG and Coll_HA is shown in Figure 14.
(b) ARS staining on OBs is performed as described in Section 3.4 “Cell Biochemistry—Cytochemical staining protocols” to be followed by stain elution: incubate each sample with 500 µL of 10% cetylpyridinium solution at room temperature for 5 min under orbital shaking. Transfer 100 µL of the solution in a 96-well plate and read the absorbance at λ 570 nm with a microplate spectrophotometer. An example of ARS eluate reading on OBs derived from the indirect co-culture with Coll_MBG and Coll_HA is shown in Figure 15.
(c) TRAP stain for PBMCs can be substituted with ELF97, a fluorescent method for TRAP-positive granule detection.
 After the removal of the cell culture medium, the samples are rinsed twice with PBS and cells are fixed with fixative solution n.2 for 20 min. Following a wash of the cell layer with PBS 1× for 5–10 min, the samples are stained with 500 µL (or a final volume able to cover the sample) of ELF97 200 µM for 15 min avoiding light exposure. After rinsing the cell layer with PBS 1× the cell layer is observed under a fluorescent microscope.
(d) Scanning electron microscopy (SEM) may be applied to observe the morphology of cells seeded on the material. After the removal of the cell culture medium, the samples are rinsed with PBS and fixed with fixative solution n.4 at 4 °C for 1 h. Following a wash with 0.1 M sodium cacodylate buffer for 15 min at room temperature, the samples are post-fixed with 1% osmium tetroxide solution at 4 °C for 1 h. After another wash with 0.1 M sodium cacodylate buffer for 15 min at room temperature, the samples are dehydrated in graded alcohol series: EtOH 25%, EtOH 50%, EtOH 70%, EtOH 80%, EtOH95% for 15 min, then EtOH 100% twice for 20 min with final dehydration twice in hexamethyldisilane (Electron Microscopy Sciences, Hatfield, PA, 16700) for 10 min at room temperature. Sputter-coated samples are examined by SEM. Representative images by SEM of cell adhesion on Coll_MBG and Coll_HA are reported in Figure 16, where OBs and PBMCs seeded on the two materials are shown.
(e) In addition, cell viability can be evaluated with live-dead assay, a fluorochrome-based method that allows the observation of viable cells seeded on the material. Briefly, after the removal of the cell culture medium and a wash with PBS 1×, the samples are incubated with the live-dead staining solution in an orbital shaker for 20 min at 37 °C, protected from light. The staining solution is prepared by mixing the two vials with 1 mL of culture medium without FBS. After rinsing with PBS, the cells are observed under an optical fluorescent microscope (Nikon Eclipse E800) and the images acquired at different magnifications using the NIS-Element imaging software BR4.00.00 (Nikon) (Figure 17).

**Figure 18:**
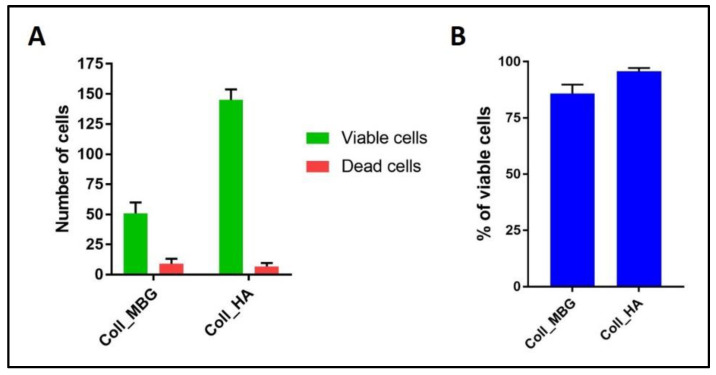
Diagram of the results at 7 days using the live-dead assay on osteoblasts seeded on the collagen-based scaffolds with nano-hydroxyapatite (Coll_HA) and collagen-based scaffolds containing mesoporous bioactive glasses (Coll_MBG). In (**A**) the number of viable and dead cells, and in (**B**) the percentage of viable cells are represented.

Two quantitative analysis on the live-dead stained OBs seeded on Coll_MBG and Coll_HA scaffolds within the indirect co-culture are shown: the number of viable (green) and dead (red) cells as counted by light microscopy (Figure 18A) and the percentage of viable cells (Figure 18B).

The quantitative evaluations were performed on four different fields at 10× magnification for each sample.

**Figure 19:**
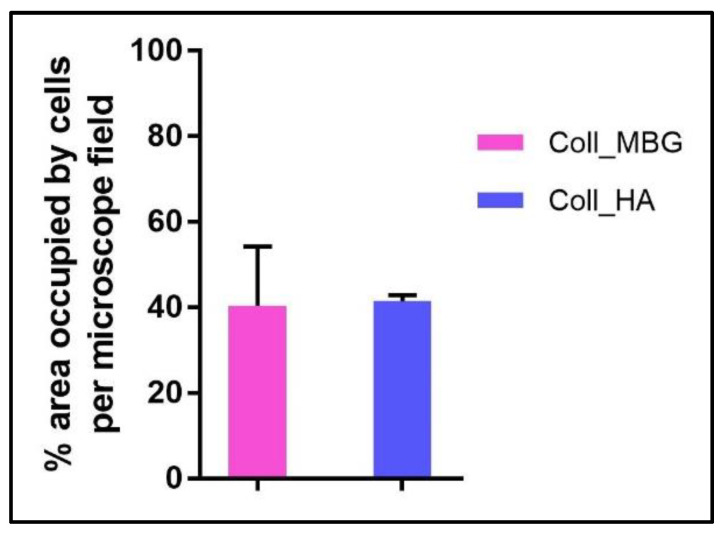
Diagram of the quantification of the area occupied by osteoblasts seeded on the collagen-based scaffolds with nano-hydroxyapatite (Coll_HA) and collagen-based scaffolds containing mesoporous bioactive glasses (Coll_MBG) and stained with the live-dead assay at 7 days.

In addition, another quantitative assay can be performed on the Live-Dead stained images, that is the evaluation of the material surface occupied by viable cells, as reported in Figure 19. For this analysis, the quantification of the green channel (viable cells) was automatically performed on three different images (20× magnification) acquired for each sample using the NIS-Element image software Advanced Research (Nikon). The threshold for the detection of the signal was set up at 7–255 with Clean 1× (Applications → Cell Counting).

### 3.6. Timing and Trouble Shooting

The timing for each step of the procedure is reported in the Table 1 below.

Possible troubleshooting concerning the procedure are reported in Table 2 below.

### 3.7. Statistical Analysis

Data are expressed as mean ± standard deviation of four replicates analyzed in two different experiments, with *p* ≤ 0.05 considered as statistically significant. The nonparametric Mann–Whitney test for unpaired data was applied, using the StatView 5.01 for Windows software (SAS Institute Inc., Cary, NC, USA).

## 4. Expected Results

### 4.1. Indirect hOB/PBMC Co-Culture

The co-culture in OM can promote the mature phenotype in comparison to the BM condition: a consistent expression of ALP, von Kossa, and Alizarin Red should be already observed after seven days in OM (Figure 4d–f) when compared to BM (Figure 4a–c). By day 14, a higher expression of ALP is detected in OM (Figure 4j) in comparison to BM (Figure 4g). At the same time, the mineral deposition as characterized by Alizarin Red and von Kossa staining may be evident, with more spots of deposited calcium in OM (Figure 4k,l) compared to BM (Figure 4h,i).

It is important to notice that for OBs, seven days of cell expansion in BM before starting the indirect co-culture systems must be considered. According to this, the 7-day co-culture timepoint corresponds to 14 days of OB culture, justifying the positive ALP staining already at an early stage of the co-culture.

Alizarin Red and von Kossa stains can be quantified by counting the number of mineral nodules: in Figure 6 representative images of ARS-stained sections used for mineral nodules quantification are shown, with the diagram reporting the data of the quantitative analysis. The indirect co-culture in OM showed a higher number of mineral nodules compared to BM.

Similarly, the quantification of mineral nodules by von Kossa stain (Figure 7) showed the same trend, with the indirect co-culture in OM displaying the highest data.

Concerning PBMCs, the differences between BM and OM in terms of osteoclast differentiation and induction of mature phenotype are not evident at seven days. PBMCs/pre-OCs cultured in BM (Figure 8a–c) may show only some early signals of differentiation like small TRAP-positive spots and the initial formation of an actin ring in comparison to OM (Figure 8d–f). At a later stage of differentiation, i.e., 14 days, clear signs of OC commitment appear, including numerous TRAP-positive multi-nucleated giant cells in OM (Figure 8j–l) with a number of nuclei per cell higher than those visible in BM (Figure 8g–i). The count of TRAP-positive multi-nucleated giant cells is reported in Figure 12. The comparison between PBMC-single control culture (Figure 9) and those in indirect co-culture (Figure 8) maintained both in BM and OM showed the higher number of TRAP-positive multi-nucleated giant cells in OM.

By phalloidin-FITC staining of actin, a remarkably higher number of large multi-nucleated cells with an evident actin ring when cultured in OM (Figure 10j–l) may be observed in comparison to BM (Figure 10g–i), especially at day 14. The quantification of actin filaments can be performed to acquire quantitative data on phalloidin-FITC-stained samples. With this aim, the software NIS-Elements Advanced Research (Nikon, Amsterdam, The Netherlands) has been applied to calculate the area occupied by positive stain in order to compare the data among the controls (single-culture of PBMCs, Figure 11) and the indirect co-cultured PBMCs exposed to BM and OM. As reported in Figure 13, the indirect co-culture in OM showed better results in comparison to control culture, confirming that the presence of osteogenically-induced OBs provide enough stimuli for OCs in the absence of M-CSF and RANKL.

Hence, the maintenance of the co-culture in OM leads to a more marked differentiation of PBMCs toward OCs at 14 days, with enhanced TRAP-positive expression and consistent actin filaments in comparison to the BM condition, therefore confirming the paracrine activity of OBs towards PBMCs.

In light of the above, the co-culture maintained in OM allows to obtain mature OBs and PBMCs, and consequently to reproduce in vitro the physiological system in which OBs and OCs exchange reciprocal signals.

When applying the indirect co-culture to biomaterials, some considerations must be taken:

- If the material is 2D and translucent, the previously reported methods can be applied without any additional precaution, as reported in Section 3.4 “Cell Biochemistry–Cytochemical Staining Protocols”.
- If the material is 3D and non-translucent, we suggest new assays to be performed (see Section 3.5 “Material Testing with the Indirect Co-Culture”), where the authors show alternative assays performed on two non-translucent collagen-based hydrogels.

ALP stain can be substituted by ALP quantification in both supernatants and cell lysates (OBs), as shown in Figure 14, where the highest value of ALP production was detected for Coll_MBG at 14 days in cell lysate.

Concerning ARS stain, once performed the staining, the dye can be eluted in order to quantify the positivity for ARS, as reported in Figure 15, where no significant differences were detected between Coll_MBG and Coll_HA.

In addition, SEM analysis can be used on cell-laden materials to observe cell morphology: the osteoblasts and PBMCs seeded onto the two materials and indirectly co-cultured are shown in Figure 16. The cell adhesion and spreading can be appreciated following SEM analysis.

Another informative test is the Live-Dead assay, which allows discriminating viable green cells from red dead cells. In Figure 17 representative images of the stain applied on Coll_MBG and Coll-HA are shown. Once performed the stain, different images are acquired, and the number of viable and dead cells (Figure 18A) as well as the percentage of viable cells can be estimated (Figure 18B), to obtain quantitative information from the assay.

Finally, the area occupied by viable cells can be estimated, to measure the cell adhesion properties of the materials, as reported in Figure 19.

The primary co-culture application of our interest concerns the screening of materials and scaffolds for BTE applications. With this aim, the material and/or scaffold can be tested with the two main players of bone formation, i.e., OBs and OCs, to provide preliminary information about the bone tissue response. The material can be seeded in turn with OBs or PBMCs, with the presence of the other cell type sharing paracrine signaling in the indirect co-culture, with co-culture maintenance for two weeks. In this way, the cell behavior of OBs and PBMCs when directly cultured on the material can be evaluated without excluding the presence of the other cell type.

### 4.2. PBMCs Single-Culture as Differentiation Control

The single culture of PBMCs supplemented with M-CSF and RANKL, which is the medium for the PBMCs’ commitment towards OCs, confirmed the formation of mature multinucleated large cells with positive staining for actin ring after 14 days of stimulation (Figure 11c,d). Osteoclasts with a clearly defined peripheral actin ring and a number of blue nuclei can be appreciated at higher magnification (Figure 11e,f). At the same time, the expression of TRAP-positive spots increased over time (Figure 9). It is important to notice that the formation of the actin ring, podosomes and other cell structures strongly depends on the material (plastic/glass/bone or dentine) the PBMCs are cultured onto.

### 4.3. OBs Single-Culture as Differentiation Control

The single-culture of OBs is performed both in BM and OM for 14 days. At 7 days of culture, the ALP positivity is detected both in BM (Figure 5a) and OM (Figure 5d). At 14 days OBs displayed a strong ALP positivity and several calcium deposits by ARS and von Kossa methods when cultured in OM (Figure 5j–l) compared to BM (Figure 5g–i).

## 5. Reagents Setup

Details on the components, concentrations, and other relevant information on the solutions used in this protocol are provided below:

- Fixative solution n.1: 3.7% paraformaldehyde (PFA) in PBS: to prepare 25 mL of fixative solution, dissolve 0.925 g of PFA (Sigma-Aldrich, P6148, Milan, Italy) in 15 mL of pre-heated (60 °C) PBS 1×, clarify with 1–2 drops of NaOH, cool in ice, measure pH 7.6, and bring to the final volume of 25 mL with PBS. Store eventually at –20 °C.
- Fixative solution n.2: 3% paraformaldehyde (PFA) with 2% sucrose (Electron Microscopy Sciences, 21,600, Hatfield, PA, USA) in PBS: to prepare 25 mL of fixative solution, dissolve 0.75 g of PFA (Sigma-Aldrich, P6148, Milan, Italy) in 15 mL of pre-heated (60 °C) PBS 1×, clarify with 1–2 drops of NaOH, cool in ice, dissolve 0.5 g of sucrose, measure pH 7.6, and bring to the final volume of 25 mL with PBS. Store eventually at –20 °C.
- Fixative solution n.3: for a final volume of 10 mL, mix 2.5 mL citrate solution, 7 mL acetone, and 0.8 mL 3.7% formaldehyde to be kept for 30 min at RT. All the previous reagents are included in the leukocyte alkaline phosphatase assay kit (Sigma-Aldrich, 86R, Milan, Italy).
- Fixative solution n.4: 2% glutaraldehyde (MERCK, Sigma-Aldrich, 4239, Milan, Italy) in 0.2 M sodium cacodylate buffer.
- 0.2 M sodium cacodylate buffer: to prepare 1000 mL, dissolve 42.8 g of sodium cacodylate (Electron Microscopy Sciences, 12310, Hatfield, PA, USA) in 800 mL of distillate water and measure pH 7.4 with the addition, if required, of HCl 37%. Bring the final volume to 1000 mL.
- 1% osmium tetroxide solution: dissolve 500 mg of osmium tetroxide (Electron Microscopy Sciences, 19100, Hatfield, PA, USA) in 25 mL of distillate water and then dilutes 1:1 with 0.2 M sodium cacodylate buffer.
- Complete culture medium for PBMCs: Dulbecco’s modification of Eagle’s medium with high glucose (Euroclone, ECB7501L, Milan, Italy) (DMEM-HG) supplemented with 10% fetal bovine serum (FBS) (Sigma-Aldrich, F7524, Milan, Italy) and 1% penicillin-streptomycin (Euroclone, ECB3001D, Milan, Italy).
- Ficoll-Paque PLUS, 1.078 g/mL (Cytiva, Sigma-Aldrich, GE17144002, Milan, Italy).
- Ca^+2^-free complete culture medium for OBs: Dulbecco’s modification of Eagle’s medium (D-MEM) (Gibco, Thermo Fisher Scientific, 31600-083, Milan, Italy) supplemented with 10% fetal bovine serum (FBS) (Sigma-Aldrich, F7524, Milan, Italy) and 1% penicillin-streptomycin (Euroclone, ECB3001D, Milan, Italy).
- Complete culture medium for OBs: Dulbecco’s modification of Eagle’s medium with low glucose (DMEM-LG) (Sigma-Aldrich, D2902, Milan, Italy) supplemented with 10% fetal bovine serum (FBS) (Sigma-Aldrich, F7524, Milan, Italy) and 1% penicillin-streptomycin (Euroclone, ECB3001D, Milan, Italy).
- Cell freezing medium: 90% of fetal bovine serum (FBS) and 10% of dimethylsulfoxide (DMSO).
- Basal medium: 1:1 of complete DMEM-LG/complete DMEM-HG medium.
- Osteogenic medium: 1:1 of complete DMEM-LG/complete DMEM-HG medium supplemented with 5 mM β-glycerophosphate (Sigma-Aldrich, G9422, Milan, Italy),10-7 M dexamethasone (Sigma-Aldrich, d-2915, Milan, Italy) and 50 µM ascorbic acid (Sigma-Aldrich, A8960, Milan, Italy).
- Differentiation medium for PBMCs: DMEM-HG supplemented with 10% fetal bovine serum (FBS) characterized (Euroclone, CHA115L, Milan, Italy), 1% penicillin-streptomycin (Euroclone, ECB3001D, Milan, Italy), containing 25 ng/mL monocyte/macrophage colony-stimulating factor (M-CSF) (Peprotech, 300-25, London, UK) and 50 ng/mL receptor activator of NF-κB ligand (RANKL) (Peprotech, 310-01, London, UK). For the first four days of culture, only M-CSF is added, while from the fourth day to the end of the culture period RANK-L is added, too.
- Trypsin-EDTA solution: trypsin 0,05%, EDTA 0,02% in PBS *w*/*o* calcium *w*/*o* magnesium *w*/*o* phenol red (Euroclone, ECB3052D, Milan, Italy).
- Alizarin Red S (ARS) staining solution: 2% (*w*/*v*) Alizarin Red S (Sigma-Aldrich, A5533, Milan, Italy) in H_2_O, pH 4.2.
- Von Kossa staining solution: solution 1 (2% silver nitrate): 2 g AgNO3 (Sigma-Aldrich, S0139, Milan, Italy) in 100 mL deionized H_2_O; solution 2 (2.5% sodium thiosulfate): 2.5 g sodium thiosulfate (Carlo Erba, 483,827, Milan, Italy) in 100 mL deionized H_2_O; solution 3 (0.33% nuclear fast red solution): 0.33 g nuclear fast red (Carlo Erba, 476,951, Milan, Italy) in 100 mL deionized H_2_O.
- Fluorescein isothiocyanate (FITC)-phalloidin (Sigma-Aldrich, P-5282, Milan, Italy) staining solution: 1 mg/mL in PBS.
- Nuclear staining: 1.25 μg/mL Hoechst 33528 (Sigma-Aldrich, 861405, Milan, Italy) in H_2_O.
- Erythrosin B staining solution 1×: 0.4 g of Erythrosin B (Sigma-Aldrich, e-9259, Milan, Italy), 0.81 g sodium chloride, 0.06 g sodium phosphate monobasic in 100 mL H_2_O. Boil the solution. Once cooled down, dilute 1:10 with dH_2_O before use.
- EDTA-based buffer for the lysis of red blood cells (RBCs): dissolve in distilled water 0.037 g/L ethylenediaminetetraacetic acid disodium salt dihydrate (EDTA) (Sigma-Aldrich, E5134, Milan, Italy), 1 g/L potassium bicarbonate (Sigma-Aldrich, P9144, Milan, Italy), 8.26 g/L ammonium chloride (Carlo Erba, 314002, Milan, Italy). Add 450 µL of this solution to the 50 µL of RBC suspension diluted in 450 µL of PBS and incubate for 10 min at 37 °C avoiding light exposure.
- Tris buffer + 0.1% Triton X-100 pH 7.5: prepare Tris buffer (10 mM, pH 7.5) containing 0.1% Triton X-100.
- 10% cetylpyridinium solution: dissolve 10 g of cetylpyridinium (Sigma-Aldrich, C0732, Milan, Italy) in 100 mL of distilled water.
- Live-Dead staining solution: mix vial 1 and vial 2 and add 1 mL of culture medium without FBS.

## 6. Conclusions and Limitations

The aim of our protocol is to describe a potential system for reproducing in vitro the bone coupling when testing new materials for BTE applications, as well as to detail the assays useful for assessing the effects of the materials on bone cells. A preliminary evaluation of the bone cells’ behavior in the presence of the material is performed through the analysis of cell morphology, adhesion, and the expression of a mature phenotype. By means of the proposed indirect co-culture, these features may be analyzed in a micro-system where the two main players of bone remodeling, i.e., osteoblasts and osteoclasts, are simultaneously present.

Our co-culture system of OBs and PBMCs is suitable for an intermediate experienced researcher; it is designed by paying attention to the costs of the laboratory equipment and reagents, while describing in detail the application of several tests for an effective evaluation, though preliminary, of new materials for BTE.

We strongly recommend the use of human cells, that is OBs from trabecular or cortical bone and PBMCs from the buffy coat, even if other cell sources may be considered. Human cells are preferred to animal-derived cells for a reliable bone replicate in vitro, even if their supply is limited, and poor availability of donor tissue together with their phenotypic heterogeneity undermines their wide application in the screening test of new materials.

The basic assays are provided, exploitable when studying the behavior of primary cells, challenged in vitro with materials for bone, such as hydrogel, electrospun materials, membranes, and so on.

The procedure for performing the assays is accurately detailed and the potential critical steps are cited, with a table dedicated to timing and troubleshooting.

By providing a set of intermediate complexity assays for the evaluation of bone materials, our protocol may be the first starting point to disclose the direct effects of the material on osteoblasts and osteoclasts, to be followed by a further second-phase advanced testing on their crosstalk.

## Figures and Tables

**Table 1:** Timing of the main steps of the protocol: osteoblasts (OBs) isolation from bone fragments and expansion, PBMCs isolation from buffy coat, material preparation, and cell seeding onto the material, cell biochemistry to evaluate cell behavior.

Step	Timing
OB isolation and expansion	3–4 weeks until confluent OB monolayer
PBMCs isolation	5 h
Material preparation and cell seeding	2 days
Cell biochemistry	1 days

**Table 2:** Troubleshooting during the different steps of the protocol.

Step	Problems	Solution
3.1	Cell aggregates are seen before seeding step in the wells	Make sure that cells are well resuspended within the culture medium
3.1	Low number of viable OBs	Check viability before starting the protocol Detach a 2nd flask of OBs
3.2 (d, f, h)	Harvesting only the white ring of PBMCs is difficult	Harvest also the plasma surrounding the white ring (see Figure 2)
3.2 (k)	PBMCs count hard to accomplish	Further dilute the cell suspension with PBS
3.2–3.3	Low seeding density of PBMCs compromises the success of the co-culture system	Process a new buffy coat if available
3.3	Difficult manipulation of the indirect co-culture set up	Remove the transwell with OBs seeded by means of sterile pliers, lay down it in a sterile multiwell plate with complete medium; then seed PBMCs at the bottom of the well previously equipped with sterile and pre-wetted TC coverslip. Lastly, insert again the transwell with OBs seeded in co-culture with the PBMCs (see Figure 3). A schematic timeline of the co-culture maintenance is shown in Figure 1.
3.5	Difficult manipulation of the material with cells seeded on the top	Remove the material from the well (to be observed under a microscope) with a spatula, lifting it from the bottom

## Data Availability

Not applicable.
